# Isolation and Characterization of n-3 Polyunsaturated Fatty Acids in *Enteromorpha prolifera* Lipids and Their Preventive Effects on Ulcerative Colitis in C57BL/6J Mice

**DOI:** 10.3390/foods13010046

**Published:** 2023-12-21

**Authors:** Haichao Wen, Pooi Mun Leong, Xincen Wang, Duo Li

**Affiliations:** 1 Institute of Nutrition and Health, Qingdao University, No. 308 Ningxia Road, Qingdao 266071, China; wenhc@qdu.edu.cn (H.W.); wangxc@qdu.edu.cn (X.W.); 2 School of Public Health, Qingdao University, No. 308 Ningxia Road, Qingdao 266071, China; leong_pm@hotmail.com

**Keywords:** *Enteromorpha prolifera*, n-3 polyunsaturated fatty acid, inflammation, ulcerative colitis, C16:4n-3, C18:4n-3, lipid metabolism

## Abstract

*Enteromorpha prolifera* (EP) is a green alga that causes green bloom worldwide. This study aimed to isolate and identify n-3 polyunsaturated fatty acids (PUFAs) from EP oil obtained via supercritical fluid extraction (SFE) and to explore its preventive effects against dextran sodium sulfate (DSS)-induced ulcerative colitis in C57BL/6J mice. In EP oil, we found the novel n-3 polyunsaturated fatty acid C16:4n-3 and two unusual fatty acids C18:4n-3 and C16:3n-3, using GC-MS. The administration of EP oil reduced histopathological of symptoms colitis and the shortening of the colon length. Pro-inflammatory cytokines of IL-6 and TNF-α in serum of EP oil treatment were lower than DSS treatment (by 37.63% and 83.52%), and *IL-6* gene expression in the colon was lower in than DSS group by 48.28%, and IL-10 in serum was higher than DSS group by 2.88-fold. Furthermore, the protein expression of p-STAT3 by the EP oil treatment was significantly reduced compared with DSS treatment group by 73.61%. Lipidomics study suggested that phosphatidylcholine and phosphatidylethanolamine were positively associated with the anti-inflammatory cytokine IL-10, while cholesteryl ester and sphingomyelin were negatively related to inflammation cytokines in the EP oil group. The present results indicated that EP oil rich in n-3 PUFA contains a novel fatty acid C16:4n-3, as well as two uncommon fatty acids C18:4n-3 and C16:3n-3. EP oil could prevent DSS-induced ulcerative colitis by regulating the JAK/STAT pathway and lipid metabolism.

## 1. Introduction

Inflammatory bowel disease (IBD) is a gastrointestinal inflammatory disease including Crohn’s disease and ulcerative colitis (UC), with an estimated prevalence of 5 million cases of UC in 2023 [1]. UC is characterized by ulcers in the rectum and colon, and the typical symptoms are rectal bleeding, tenesmus, increased stool frequency or episodes of diarrhea, urgent bowel movements, and cramp-like abdominal pain [2]. The cause of UC is complex and not completely clear, and might be related to many factors like genetic, microbiota, immune system, stress, and diet [3]. Risk factors, including a Westernized diet characterized by a high intake of saturated fats and refined sugar, smoking, stress, and antibiotics can increase the incidence of UC [4]. Medications such as 5-aminosalicylic acids (sulfasalazine, mesalamine, balsalazide and olsalazine), corticosteroids (prednisone and budesonide), thiopurines, immunomodulators, and other biologics are typically used for the maintenance of UC remission [2]. However, these treatments are not suitable for long-term use due to their adverse effects such as bone marrow and liver toxicity, pancreatitis, lymphoma, osteoporosis, depression, type 2 diabetes mellitus, and herpes zoster [2]. Recently, researchers have looked at specific dietary interventions like the Mediterranean diet and anti-inflammatory diets to protect intestinal immune homeostasis [5].

Marine-derived n-3 polyunsaturated fatty acids (PUFAs) like eicosapentaenoic acid (EPA, C20:5n-3), docosapentaenoic acid (DPA, C22:5n-3), and docosahexaenoic acid (DHA, C22:6n-3) and their precursors like α-linolenic acid (ALA, C18:3n-3) and stearidonic acid (SDA, C18:4n-3) may influence the inflammatory state of endothelial cells [6]. Especially, EPA and DHA may protect against TNBS- or DSS- induced UC in vivo, but ALA might not reduce the development of UC [6]. Besides, n-3 PUFAs like EPA, DPA, and DHA could be converted to specialized pro-resolving mediators (SPMs) such as resolvins, protectins, and maresins, which may play important roles in the inflammatory response [7]. However, it is still needed to investigate the potential SPMs of C18:4n-3 and the beneficial effects on IBD. Moreover, fish oil is not recommended for vegetarians and people with fish allergies those that dislike its strong odor and flavor. In this case, algae oil may be a potential alternative PUFA. 

*Enteromorpha prolifera* (EP) is a green alga distributed globally that is used as food and traditional medicine in China and Japan [8]. In recent decades, EP bloom has caused large-scale green tides, which have led to serious problems for marine aquaculture and the environment [9]. EP is rich in proteins, sulfated polysaccharides, unsaturated fatty acids, and mineral elements like ferrum and calcium. Many studies focus on polysaccharides, which have biological effects such as against oxidative stress, insulin resistance, inflammation, and cancer [8,10]. In addition, the ethanol extract of EP was active against lipid accumulation, inflammatory and oxidative stress on high-fat diet-fed mice [11]. However, the composition of the fatty acids in EP oil and the functional properties are less well-studied. Although the lipids account for less than 5% of green algae, algae oil is usually rich in PUFAs, which may be effective against inflammation, cardiovascular disease, and metabolic disorders [12]. 

Consequently, the aim of the study was to investigate the fatty acids of EP oil and the anti-inflammatory effect. EP oil was isolated by supercritical fluid extraction and the fatty acid composition of GC-MS was analyzed. We evaluated the mechanisms of anti-inflammatory activity and potential metabolite biomarkers in DSS-induced colitis in C57BL/6J mice.

## 2. Materials and Methods

### 2.1. Plant Material and Reagents

*Enteromorpha prolifera* was collected from the offshore of Qingdao, Shandong, China (36°05′ N, 120°30′ E), dried at 60 °C, and then ground into powder. The molecular mass of dextran sodium sulfate (DSS) was 36~50 kDa (Yeasen Biotechnology (Shanghai) Co., Ltd., Shanghai, China). Acetonitrile, methanol, formic acid, and ammonium acetate were provided by Merck (Darmstadt, Germany) and the other reagents were of analytical grade. 

### 2.2. Isolation of Enteromorpha prolifera Oil by SFE

Lipids were isolated from EP following the previous procedure detailed in Yang et al. [13]. Supercritical fluid extraction (SFE) was carried out using a supercritical fluid extraction system (HA220-40-11) (Jiangsu Huaan Scientific Research Devices Co., Ltd., Nantong, China). EP powder was mixed with 95% ethanol (4:1 *w*/*w*) and fed into a 10 L extraction vessel. The temperatures of separator I and II were 55 °C and 37 °C whereas the pressures were 8 and 5 MPa, respectively. The temperature and pressure of the extractor were 45 °C and 28 MPa. EP oil was collected from the two separators every 45 min and then combined.

### 2.3. Fatty Acid Methyl Esters and GC-MS Analysis

Lipids from samples were extracted by chloroform and methanol (2:1, *v*/*v*). Then, lipids were transesterified to fatty acid methyl esters (FAMEs) by the previous procedure [14]. Briefly, 3 mL 0.9 mol/L H_2_SO_4_/methanol and 1 mL methylbenzene were added to 1 mL of the sample. The tubes were capped, placed in a 70 °C water bath for 2 h, and shaken every 30 min. Then, 2 mL hexane and 2 mL water were added to the sample and centrifuged at 2000 rpm for 10 min. The oil suspension was filtered using a Supelclean LC-Si SPE silica column (505048, Supelo, Sigma-Aldrich (Shanghai) Trading Co., Ltd., Shanghai, China) and the lipid extract was dried using gaseous nitrogen. 

GC-MS analysis was performed with an Agilent 7000D GC and 5973 (EI) MS detector (Agilent Technologies, Palo Alto, CA, USA). Agilent MassHunter Qualitative Analysis 10.0 software was used for data processing [15]. The flow rate was 1 mL/min and an HP-INNOWax column (30 m × 250 μm × 0.25 μm, Agilent 19091N-133I) was used. Program was set for 5 min from 160 °C at 20 °C/min; then 12 min from 180 °C at 20 °C/min; 8 min from 200 °C at 20 °C; 11 min from 205 °C at 20 °C; 5 min from 280 °C at 6 °C. The GC was operated in split injection mode and split ratio was 10:1 at a split flow rate of 10 mL/min with helium as the carrier gas (15 mL/min). Injection volume was 1 μL and inlet temperature was 230 °C. Detector temperature was set at 320 °C and scanning range was from 300~800 *m*/*z*. Peaks were identified by retention time and characteristic ions by comparison with a standard 37-component FAME mix (Sigma-Aldrich (Shanghai) Trading Co., Ltd., Shanghai, China) and the NIST 11 library. 

### 2.4. Animals and Treatment

Male C57BL/6J mice (20~24 g) were provided by Beijing Vital River Laboratory Animal Technology Co., Ltd. (Beijing, China). Ethical approval was obtained from the Qingdao University Laboratory Animal Welfare Ethics Committee (No. 20211231C576020220111070). All mice were housed at 22 ± 2 °C with 50% ± 5% relative humidity and a 12 h light/dark cycle light. After one week of acclimation, the mice were randomly allocated (6 mice per group) to the four groups. All mice had free access to a standard diet (AIN-93M) and were given oil by intragastric administration, as follows: (1) control (olive oil, 0.1 mL/20 g), (2) DSS (olive oil, 0.1 mL/20 g), (3) EP oil (EP oil, 0.1 mL/20 g), (4) SASP (salicylazosulphapyridine, 100 mg/kg and olive oil, 0.1 mL/20 g) [16]. The control group was given sterile tap water, whereas the other three groups were provided 2.0% DSS in the drinking water on days 1~4, days 14~18, and days 29~33 to induce chronic colitis [17,18]. Body weight was recorded daily, and mice were fasted overnight at the end of experiment and sacrificed after anesthesia. Blood was obtained from the orbital venous plexus bleeding, and after allowed to stand for 30 min, serum was collected by centrifuging at 2000× *g* for 15 min in 4 °C.

### 2.5. Histopathological Examination

Colon tissues were formalin-fixed and paraffin embedded, and sections were sliced into 5 μm slices stained with either hematoxylin and eosin (H&E) or Alcian blue/periodic acid–Schiff (AB-PAS). The sections were visualized under a Nikon light microscope (Tokyo, Japan). Histomorphological scores of 0~4 were given to the slides based on the degree of mucosal damage and inflammatory cell infiltration in the colon. The following point system was used as a guide when assessing the slides: 0, intact crypt with no inflammatory cell infiltration; 1, minimal loss of goblet cells and inflammatory cell immersion; 2, extensive loss of goblet cells and inflammatory infiltration in the mucosa; 3, extensive loss of goblet cells, minimal loss of crypts, and inflammation continuously distributed in the mucosa; 4, extensive loss of crypts and full-thickness inflammatory cells seriously infiltrated [19].

### 2.6. Serum Inflammatory Cytokines

Cytokines in serum were quantified using the enzyme-linked immunosorbent assay (ELISA) kits for mouse IL-6, IL-10, and TNF-α (Thermo Fisher Scientific Co., Ltd., Shanghai, China). Protein concentrations were measured by BCA protein assay kit (Dalian Meilun Biotechnology Co., Ltd., Dalian, China). 

### 2.7. Quantitative Real-Time Polymerase Chain Reaction (qRT-PCR) Assay

Total RNA of colon tissue was extracted using Trizol, and reverse transcription was performed by cDNA synthesis kit (Yeasen Biotechnology (Shanghai) Co., Ltd., Shanghai, China). The amplification and detection were performed on a QuantStudio 1 Real-Time PCR System, and data were analyzed using 2^−ΔΔCT^ method by Quantstudio™ Design and Analysis Software v1.5.1 (Applied Biosystems, Foster City, CA, USA). Primer sequences are shown in Table 1. 

### 2.8. Western Blotting

Proteins from colon tissue were separated by SDS-PAGE, and transferred to 0.45 μm PVDF membranes that were blocked in 5% BSA. Primary antibodies were incubated overnight at 4 °C: anti-STAT3 (signal transducer and activator of transcription 3, STAT3) (1:1000, ab68153; Abcam, Cambridge, MA, USA), anti-p-STAT3 (phosphor Y705) (1:2000, ab76315; Abcam, USA), and anti-β-actin (1:2000, ab8227; Abcam, USA). The membranes were washed and incubated with horseradish peroxidase-labeled secondary antibody at room temperature for 1 h. Images were visualized with using ECL reagent on a Tanon-5200 Multi automatic image analyzer (Tanon, Shanghai, China) and the intensity was calculated with ImageJ 1.45s software.

### 2.9. Hepatic Lipidomics Analysis

Hepatic lipid extraction was processed by the Folch method with minor modifications [20]. Livers (liver:deionized water 1:10, *v*/*v*) were homogenized using tissue homogenizer, and liver homogenate was added to chloroform/methanol (2:1, *v*/*v*) in a 1:9 ratio, and then centrifuged at 10,000 rpm for 10 min. The organic phase was collected and dried using nitrogen. Isopropanol (200 μL) was added to redissolve, and the supernatant obtained after centrifuging at 10,000 rpm for 10 min was filtered for lipidomics analysis. Quality control (QC) samples of 10 μL were collected for each sample.

Hepatic lipidomics was performed using Agilent Technologies 6530C Q-TOF LC/MS in positive ESI detection mode. The ACQUITY UPLC BEHC18 column (2.1 mm × 100 mm, 1.7 μm) was used and the flow rate was 0.3 mL/min. Mobile phase A was acetonitrile: water (60:40, *v*/*v*) containing 5 mM ammonium acetate, and mobile phase B was isopropanol alcohol:acetonitrile (90:10, *v*/*v*). The following elution gradient was applied: initial gradient of 10% mobile phase B for 2 min, followed by a linear increase to 60% B in 6 min, which was held for 3 min, increased to 75% at 13 min, 78% at 17 min, and 99% at 19 min, which was then held for 6 min, followed by a decrease to 10% at 26 min to equilibrate the system [21]. Injection volume was 2 μL, and the other parameters set as follows: column temperature: 40 °C, scan range: 50~1000 *m*/*z*, capillary voltage: 3 kV, cone hole voltage: 40 V, capillary temperature: 320 °C, auxiliary gas heater temperature: 350 °C. All samples were randomly injected and QC samples were injected every 10 samples. 

Hepatic lipidomic data were converted by Abf converter v1.8 software; lipid metabolites were integrated by MS-FLO (https://msflo.fiehnlab.ucdavis.edu, accessed on 21 November 2023) and identified based on MS-DIAL [22]. The MS data were matched by lipidBlast database by comparing with retention times, *m*/*z* value, peak area, and ion intensities. The metabolites were analyzed using MetaboAnalyst 4.0 software (Wishart Research Group, University of Alberta, Edmonton, AB, Canada) and human Metabolome Database (HMDB) [23]. 

### 2.10. Statistical Analysis

Data in histograms were shown as the mean ± standard deviation. The results were statistically analyzed by one-way analysis of variance (ANOVA) for Duncan’s test using IBM SPSS Statistics 20 and GraphPad Prism version 8 (GraphPad Software, San Diego, CA, USA). Lipidomic data were selected by significant features (*p* < 0.01), fold-change > 2, *p*-value < 0.05, and VIP scores > 2.0. 

## 3. Results and Discussion

### 3.1. Identification of Fatty Acids of Enteromorpha prolifera Oil Extracted by SFE

EP oil was extracted by SFE, and the fatty acid composition was analyzed by GC-MS (Table 2). Linoleic acid and α-linolenic acid, which are essential fatty acids for human health, were found to be the predominant fatty acids in EP oil, accounting for 13.14% and 16.41%, respectively.

**Figure 1:**
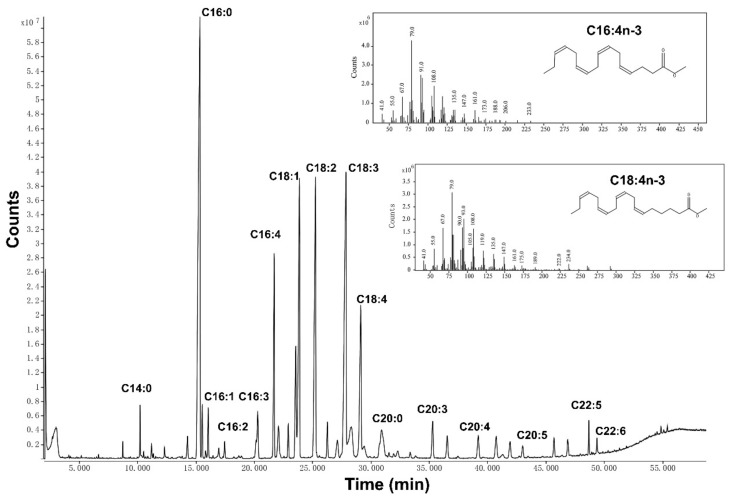
Representative GC-MS chromatogram of EP oil with the major peak and the abundance of m/z for C16:4n-3 and C18:4n-3.

The SFA:MUFA:PUFA ratio was nearly 3:2:5, whereby 32.17% of the total fatty acids were n-3 PUFAs. C16:3n-3, C16:4n-3, and C18:4n-3 of n-3 PUFAs were identified from EP oil, and C16:4n-3 and C18:4n-3 were 5.96% and 6.23% of the total fatty acids. Results showed that EP oil was rich in C16 and C18 fatty acids, especially two unusual fatty acids of C16:4n-3 and C18:4n-3 (Figure 1). 

C16 and C18 PUFAs were the main fatty acids in green algae, and these can be used to synthesize EPA and DHA in both algae and mammals [24]. ALA needs the Δ^6^-desaturase to transform to C18:4n-3, which is a rate-limiting step, then converted to EPA via elongase and Δ^5^-desaturase. On the other hand, C16:3n-3 converts to C16:4n-3 via Δ^15^-desaturase, and then transforms to C18:4n-3 via elongase, which is more easily converted to EPA and DHA. The fatty acid composition of EP oil was similar to the green seaweeds such as Ulva species [25]. In addition, C16 n-3 PUFAs can be found in *Karenia mikimotoi* and *Euphausia pacifica*, which play important roles in the marine food chain [26,27]. 

Interestingly, owing to the high n-6/n-3 fatty acid ratio (20~50:1) of modern diets related to chronic disease, the ratio of EP oil was 0.53:1, which indicates that EP oil may balance the high n-6/n-3 ratio diet [28]. Results showed that EP oil rich in n-3 PUFA contained a novel fatty acid, C16:4n-3, as well as two uncommon fatty acids, C18:4n-3 and C16:3n-3.

### 3.2. Impact of Enteromorpha prolifera Oil on DSS-Induced Mice

**Figure 2:**
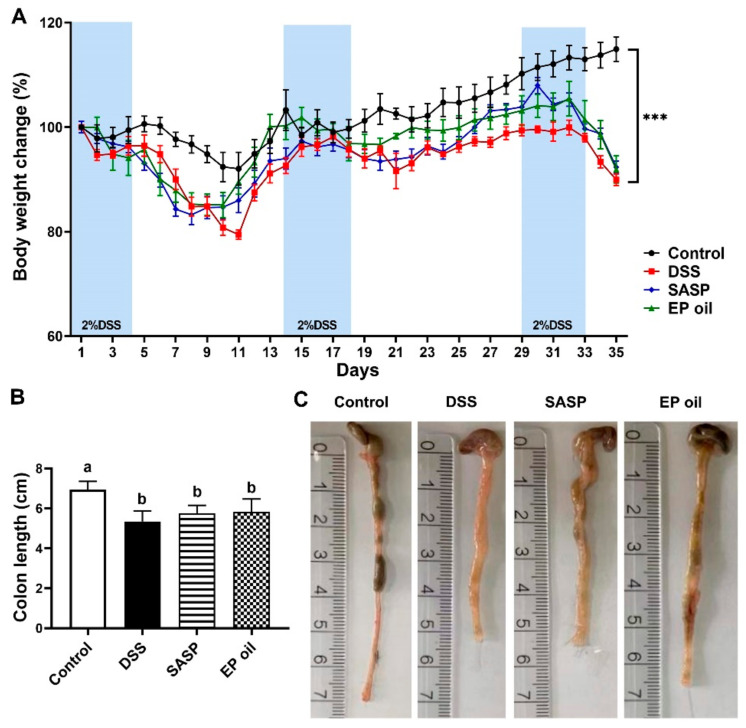
Effect of EP oil on DSS-treated mice on (**A**) body weight change; (**B**) colon length; and (**C**) representative images of the colon length. *** *p* < 0.001 compared with the DSS group. Lowercase letters in histograms indicate *p* < 0.05 according to Duncan’s test.

The protective effect of EP oil against UC was investigated in C57BL/6J mice exposed to 2% DSS for three times to induce chronic UC in a mouse model. At the end, the body weight of mice in all experimental groups exposed to DSS was significantly lower in the than control group (Figure 2A). The colonic length in the DSS-treated groups was shortened compared with the control group and clinical manifestations of DSS-induced UC including diarrhea and blood in the feces and anus was observed (Figure 2B). EP oil alleviated edema in the colon relative to the DSS group (Figure 2C).

The results showed that EP oil alleviated DSS damage to the colon, whereas the DSS group fed with olive oil rich in C18:1n-9 experienced no amelioration of UC. Another study also reported that olive oil and flaxseed oil rich in C18:3n-3 had no preventive effect on DSS-induced acute UC [29]. These results suggested that EP oil rich in C16:4n-3, C18:4n-3, and EPA could potentially attenuate DSS-induced colitis in mice.

### 3.3. Effect of Enteromorpha prolifera Oil on Colon Histomorphology 

**Figure 3:**
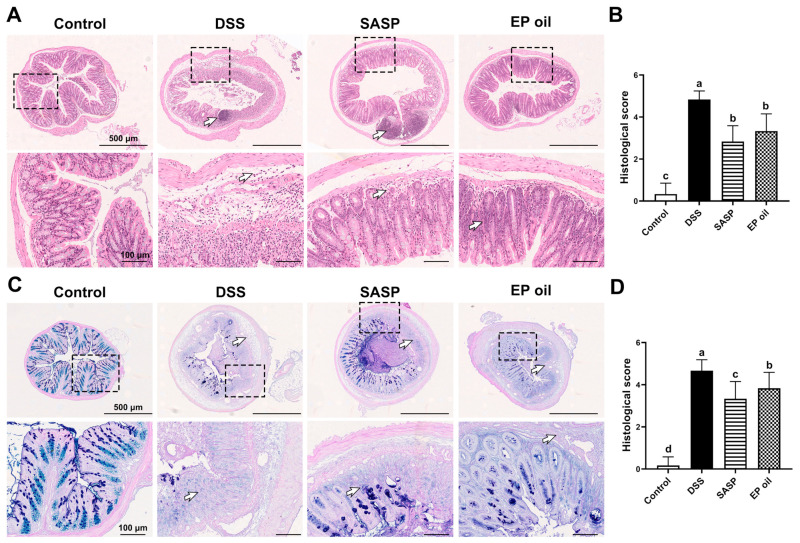
Effect of EP oil on DSS-induced mice colon by H&E and AB-PAS. (**A**) Histological staining of H&E (50× and 200×). (**B**) Histological scores of H&E. (**C**) Histological staining of AB-PAS (50× and 200×). (**D**) Histological scores of AB-PAS. White arrow pointed edema, cell infiltration, distorted crypt, or destructed epithelial barrier. Lowercase letters in histograms represent *p* < 0.05 according to Duncan’s test.

H&E staining of colon segments in the DSS group showed severe colon damage, characterized by edema, cell infiltration, distorted crypt, and destructed epithelial barrier as the white arrow pointed (Figure 3A). Histomorphological observations showed that 2% DSS induced a severe chronic UC mice model. EP oil-fed mice showed little basal plasmacytosis and goblet cell depletion compared with DSS-induced mice. Moreover, AB-PAS staining results showed neatly arranged goblet cells and less edema in the mucus membrane in EP oil and SASP groups compared to DSS mice (Figure 3C). EP oil and SASP treatment also resulted in lower histomorphological scores than the DSS group (Figure 3B,D). These results showed that EP oil could alleviate the symptoms of DSS-induced colitis in colon.

### 3.4. Anti-Inflammatory Effects of Enteromorpha prolifera Oil on DSS-Induced Mice

**Figure 4:**
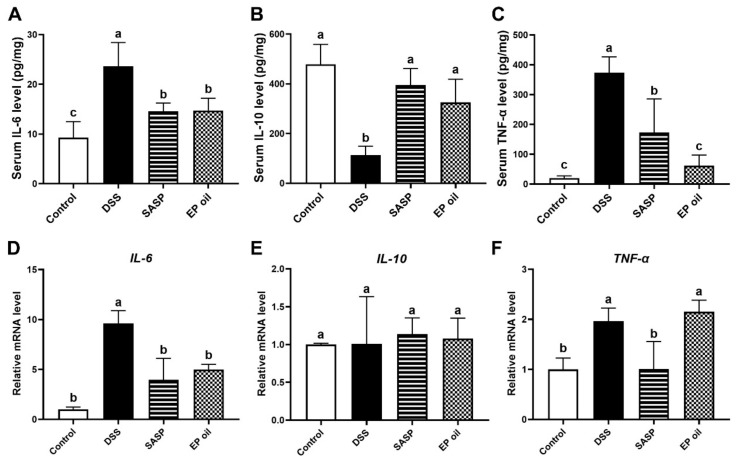
Anti-inflammatory activity of EP oil on cytokines by ELISA. (**A**) IL-6, (**B**) IL-10, and (**C**) TNF-α. The mRNA expression of cytokines (**D**) IL-6, (**E**) IL-10, and (**F**) TNF-α. Lowercase letters in histograms represent *p* < 0.05 according to Duncan’s test.

The anti-inflammatory activities of EP oil were investigated by measuring serum cytokines using ELISA, and the expression of these cytokines genes in the colon tissue was tested by qRT-PCR. IL-6 and TNF-α levels in the DSS group were significantly higher than in the other groups, and those of IL-10 were lower (*p* < 0.05). Mice treated with EP oil had significantly lower serum levels of IL-6 and TNF-α, by 37.63% and 83.52% respectively, when compared to the DSS group mice. Mice in EP and SASP groups had significantly higher expression of IL-10 than the DSS group, by 2.88-fold (*p* < 0.05) (Figure 4A–C). These results showed that inflammatory cytokines (IL-6 and TNF-α) increased after DSS treatment, whereas EP oil and SASP suppressed the secretion of pro-inflammatory cytokines and improved the secretion of anti-inflammatory cytokines. 

Meanwhile, EP oil and SASP significantly blocked *IL-6* expression compared with the DSS group by 48.28% and 58.83% (*p* < 0.05) (Figure 4D). This was especially evident in SASP-treated mice, although there was no differences in mRNA levels of *TNF-α* between the EP oil group and DSS group (*p* > 0.05) (Figure 4F). There were no differences in mRNA expression of *IL-10* in colon between all groups (Figure 4E). This observation is consistent with previous studies that n-3 PUFA alleviated inflammatory and decreased serum *TNF-α* through the formation of resolvins and protectins in fat-1 transgenic mice [30]. These showed that EP oil may play a role in cytokine modulation and exhibit anti-inflammatory activity by inhibiting *IL-6*. 

### 3.5. Enteromorpha prolifera Oil Inhibited Inflammation by JAK/STAT Signaling Pathway

**Figure 5:**
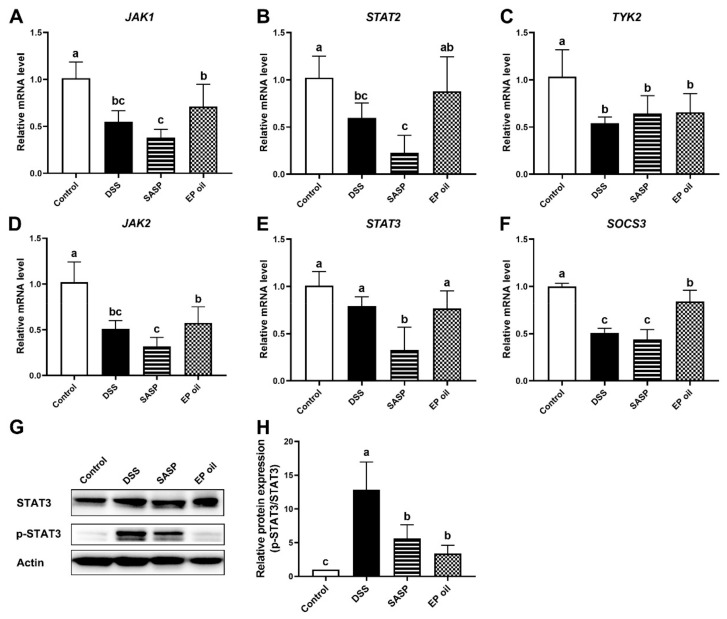
EP oil inhibited inflammation by JAK/STAT signaling pathway. (**A**) JAK1, (**B**) STAT2, (**C**) SOCS3, (**D**) JAK2, (**E**) STAT3, (**F**) TYK2 mRNA expression of EP oil, fish oil, and flaxseed oil. (**G**) Representative Western blotting images depicting STAT3 and p-STAT3, and (**H**) STAT3 and p-STAT3 protein level in colon. Lowercase letters in histograms represent *p* < 0.05 according to Duncan’s test.

The JAK/STAT signaling pathway is involved in the pathology of IBD [28]. To investigate the mechanism of EP oil on DSS-induced mice, we tested the expression of key mRNAs of JAK/STAT signaling pathway (Figure 5). *JAK1*, *JAK2*, *STAT2*, *SOCS3,* and *TYK2* mRNA levels in the DSS treatment group were lower than in the control group (*p* < 0.05). *JAK1*, *JAK2*, *STAT2*, *STAT3*, and *SOCS3* expression in mice fed SASP were lower than in other groups (*p* < 0.05). The expression of *JAK1*, *STAT2*, and *SOCS3* in the EP oil group was significantly higher than in the DSS group (*p* < 0.05). Moreover, the protein expression levels of p-STAT3 in the colon were significantly decreased following EP oil treatment, by 73.61% compared to the DSS group (Figure 5G,H). This suggests that EP oil may modulate the JAK/STAT pathway. 

Very few studies have investigated the bioactivity of C18:4n-3 and C16:4n-3 in EP oil. Some studies reported that C18:4n-3 and C16:4n-3 from *Undaria pinnatifida* and *Ulva pertusa* may convert to EPA, DHA, and pro-resolving lipid mediators, which could exert anti-inflammatory activity [31,32]. Algal oil and marine-derived bioactive compounds alleviated DSS-induced inflammation and colitis by altering the gut microbiota [12,33]. Moreover, specialized pro-resolving mediators such as resolvins, protectins, and mavrins were synthesized from EPA and DHA, and were shown to improve colon histology and reduce pro-inflammatory cytokines in the JAK/STAT signaling pathway [34,35]. This study indicated that EP oil may protect against UC via the modulation of the JAK/STAT signaling pathway. 

### 3.6. Effect of Enteromorpha prolifera Oil on Hepatic Lipid Metabolism

**Figure 6:**
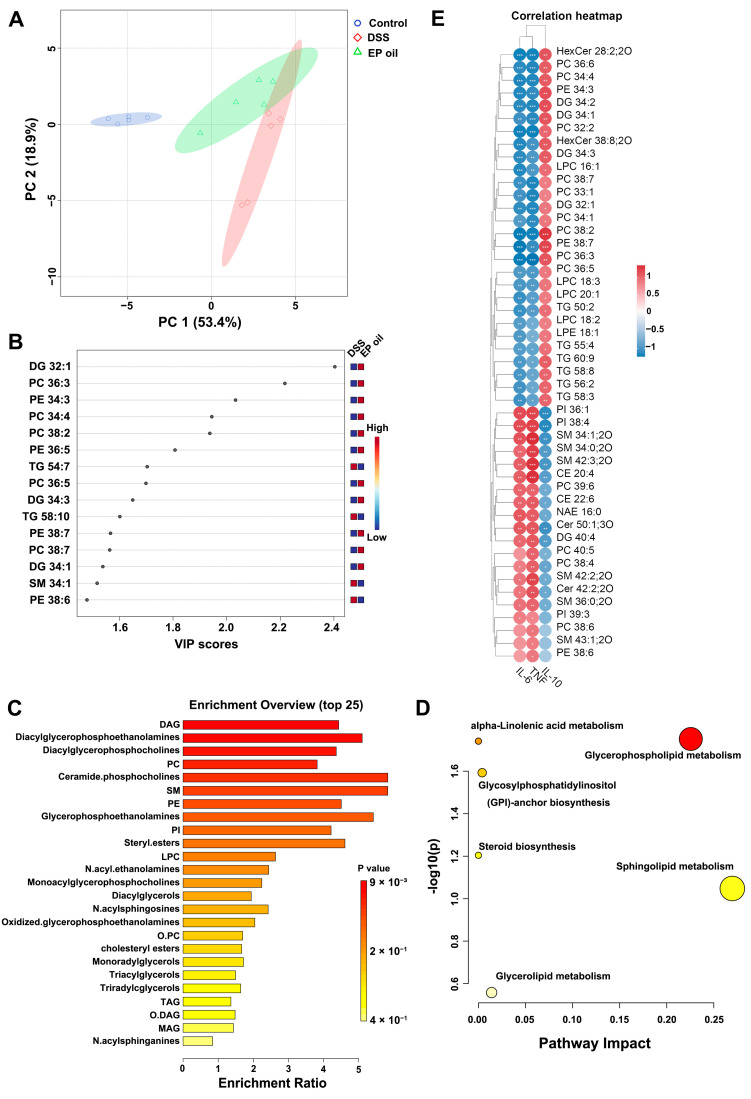
EP oil influenced lipid metabolism in liver: (**A**) PCA score plots (*n* = 5); (**B**) VIP map of PLS-DA between DSS and EP oil group; (**C**) enrichment of lipid metabolites; (**D**) pathway impact of lipid metabolites; (**E**) heatmap of association between cytokines and significantly changed metabolites.

To investigate effect of EP oil on hepatic lipid metabolism in DSS-treated mice, lipidomics were investigated using an untargeted metabolomics approach. Potential functional biomarkers of lipid metabolites were analyzed in the control, DSS, and EP oil groups. In the liver, 122 lipids were identified and a PCA-based clustering approach revealed a strong segregation with PC1 (53.4%) and PC2 (18.9%) (Figure 6A). 

PLS-DA analysis assessed the changes in lipid metabolites after DSS-induced colitis and EP oil treatment (Figure S1). VIP (variable importance in projection) for the top 15 significant lipid metabolites were selected in the EP oil and DSS groups (Figure 6B). The lipid metabolites including diacylglycerol (DG), phosphatidylcholine (PC), and phosphatidylethanolamine (PE) with polyunsaturated fatty acids were higher in the EP oil group than in the DSS group. The DSS and control groups were separated by PLS-DA, and the VIP scores of the DSS group were clustered with DG, ceramide (Cer), glycerophospholipids (PI), and sphingomyelin (SM) (Figure S1C). 

The heatmap showed that mice in the DSS group had significantly lower triacylglycerol expression (TG) and significantly higher expression of PI, SM, cholesteryl ester (CE), PC, and PE compared to the control group (Figure S2). Significantly changed lipid metabolites were selected for by the volcano plot based on fold-change and *t* tests (Figure S3). There were clear differences in the expression of lipid metabolites including DG 32:1, DG 34:3, PC 36:3, PE 34:3, PC34:4, PC36:5, and PC 38:2 between the DSS group and the EP oil group. 

Enrichment analysis showed that PI, ceramide phosphocholines, SM, DG, and PC were associated with DSS-induced colitis (Figure S4). EP oil had an effect on the expression of metabolites including phospholipids, SM, ceramide phosphocholines, PC, PE, PI, and steryl esters (Figure 6C). Furthermore, pathway analysis showed that glycerophospholipid metabolism, sphingolipid metabolism, glycerolipid metabolism, steroid biosynthesis, glycosylphosphatidylinositol (GPI) biosynthesis were associated with the EP oil treatment group and the DSS-induced colitis group (Figure 6D and Figure S5).

The correlations of lipid metabolites and cytokines were analyzed by Pearson correlation test (Figure 6E). In total, 48 lipid metabolites were significantly correlated with inflammatory cytokines. HexCer, PC, PE, LPC, and TG were related to IL-10, particularly DG 32:1, PC 36:3, PE 34:3, PC34:4, and PC 38:2. Moreover, PI, SM, CE, and Cer were found to be significantly associated with IL-6 and TNF-α. The above results suggested that lipid metabolites containing long-chain and unsaturated fatty acids exerted stronger anti-inflammatory effects. 

Abnormal lipid metabolism have associated with the risk of intestinal inflammatory; notably, PC, Cer, and SM were most significantly changed [36,37]. Lipidomics analysis showed that DSS induced changes in hepatic lipid metabolite composition. TGs in the DSS group were also significantly lower than in the control group. LPC, PC (16:0/20:4), TG (16:0/18:0/18:1), SM (18:2/24:0), TG (14:0/16:0/18:2), TG (18:1/18:2/20:4), CE (14:1), PE (O-16:0/20:4), SM (d18:1/21:0) and sitosterol sulfate are biomarkers of UC and Crohn’s disease [38,39]. Mice in the DSS group had increased PI, SM, CE, LPC, and PC, which was correlated with IL-6 and TNF-α [38,40,41]. Sphingolipids, including hexosylceramide (HexCer 38:8), PC36:6, PC36:3, PC38:2, PE38:7, PC38:7, and TG58:8, which contain long-chain polyunsaturated fatty acids had a positive association with IL-10. These results indicate that EP oil has the potential ability to regulate lipid metabolism and alleviate inflammation owing to its content of C16 and C18 n-3 PUFAs. 

## 4. Conclusions

In summary, our findings that highlight EP oil is rich in C16 and C18 n-3 PUFAs, especially C16:3n-3, C16:4n-3, and C18:4n-3, which could alleviate DSS-induced colitis and regulate lipid metabolism. As the monomer compounds of C16:3n-3, C16:4n-3, and C18:4n-3 were difficult to prepare, further study is needed to clarify the anti-inflammatory mechanisms of C16:3n-3, C16:4n-3, and C18:4n-3. These findings suggest that *Enteromorpha prolifera* may be a promising new method of dietary prevention for UC treatment.

## Figures and Tables

**Table 1:** Primer sequences for qRT-PCR analysis.

Primer	Forward (5′-3′)	Reverse (5′-3′)
IL-6	CTGCAAGAGACTTCCATCCAG	AGTGGTATAGACAGGTCTGTTGG
IL-10	GCTGGACAACATACTGCTAACC	ATTTCCGATAAGGCTTGGCAA
TNF-α	CTGAACTTCGGGGTGATCGG	GGCTTGTCACTCGAATTTTGAGA
JAK1	AGTGCAGTATCTCTCCTCTCTG	GATTCGGTTCGGAGCGTACC
JAK2	GGAATGGCCTGCCTTACAATG	TGGCTCTATCTGCTTCACAGAAT
STAT2	GTTACACCAGGTCTACTCACAGA	TGGTCTTCAATCCAGGTAGCC
STAT3	AATATAGCCGATTCCTGCAAGAG	TGGCTTCTCAAGATACCTGCTC
TYK2	TGCATCCACATCGCACACAA	CTCCTGGGGATTCATGCCA
SOCS3	TGCGCCTCAAGACCTTCAG	GCTCCAGTAGAATCCGCTCTC

**Table 2:** The fatty acid composition of *Enteromorpha prolifera* oil.

Fatty Acids	Fatty Acid	Formula	Methyl Ester (%)
C14:0	Myristic acid	C_14_H_28_O_2_	0.78 ± 0.09
C16:0	Palmitic acid	C_16_H_32_O_2_	27.56 ± 1.41
C18:0	Stearic acid	C_18_H_36_O_2_	0.79 ± 0.27
C20:0	Eicosanoic acid	C_20_H_40_O_2_	0.48 ± 0.40
SFA			29.61 ± 2.16
C16:1	Palmitoleic acid	C_16_H_30_O_2_	1.62 ± 0.43
C17:1	cis-10-Heptadecenoic acid	C_17_H_32_O_2_	0.53 ± 0.02
C18:1n-9	Oleic acid	C_18_H_34_O_2_	7.11 ± 5.67
C18:1n-7	Oleic acid	C_18_H_34_O_2_	11.39 ± 0.38
C22:1n-9	13-Docosenoic acid	C_22_H_42_O_2_	0.99 ± 0.17
MUFA			21.65 ± 6.68
C16:2n-7	9,12-Hexadecadienoic acid	C_16_H_28_O_2_	0.46 ± 0.04
C16:3n-3	7,10,13-Hexadecatrienoic acid	C_16_H_26_O_2_	1.80 ± 0.38
C16:4n-3	6Z,9Z,12Z,15Z-hexadecatetraenoic acid	C_16_H_24_O_2_	5.96 ± 0.71
C18:2n-6	Linoleic acid	C_18_H_32_O_2_	13.14 ± 0.63
C18:3n-6	γ-Linolenic acid	C_18_H_30_O_2_	0.95 ± 0.07
C18:3n-3	α-Linolenic acid	C_18_H_30_O_2_	16.41 ± 1.11
C18:4n-3	Stearidonic acid, SDA	C_18_H_28_O_2_	6.23 ± 0.91
C20:3n-6	Eicosatrienoic acid	C_20_H_34_O_2_	1.45 ± 0.09
C20:4n-6	Eicosatetraenoic acid	C_20_H_32_O_2_	0.92 ± 0.10
C20:5n-3	Eicosapentaenoic acid, EPA	C_20_H_30_O_2_	0.98 ± 0.13
C22:4n-6	Docosatetraenoic acid	C_22_H_36_O_2_	0.48 ± 0.19
C22:5n-3	Docosapentaenoic acid, DPA	C_22_H_34_O_2_	0.63 ± 0.14
C22:6n-3	Docohexaenoic acid, DHA	C_22_H_32_O_2_	0.31 ± 0.10
PUFA			49.72 ± 4.61
PUFAs n-6			17.09 ± 1.03
PUFAs n-3			32.17 ± 3.53
Ratio n-6/n-3			0.53

SFA, saturated fatty acid; MUFA, monounsaturated fatty acid; PUFA, polyunsaturated fatty acid.

## Data Availability

Data is contained within the article or Supplementary Materials.

## Supplementary Materials

The following supporting information can be downloaded at: https://www.mdpi.com/article/10.3390/foods13010046/s1, Figure S1: PLS-DA scores plot of (A) control and DSS groups and (B) EP oil and DSS groups. (C) VIP of PLS-DA between control and DSS groups; Figure S2: Heat map of (A) control and DSS groups and (B) EP oil and DSS groups; Figure S3: Volcano plot of (A) control and DSS groups and (B) EP oil and DSS groups; Figure S4: Lipid metabolite enrichment analysis between control and DSS groups; Figure S5: Metabolism pathway analysis of lipid metabolites between control and DSS groups.
