# Supplementary material for: Isolation and Characterization of n-3 Polyunsaturated Fatty Acids in Enteromorpha prolifera Lipids and Their Preventive Effects on Ulcerative Colitis in C57BL/6J Mice
Source: Foods. 2023 Dec 21;13(1):46. doi: 10.3390/foods13010046 (PMC10778640; doi:10.3390/foods13010046)

Supplementary data

The following are the Supplementary data to this article:

Figure S1 PLS-DA scores plot of (A) control and DSS groups and (B) EP oil and DSS group.

(C) VIP of PLS-DA between control and DSS groups.

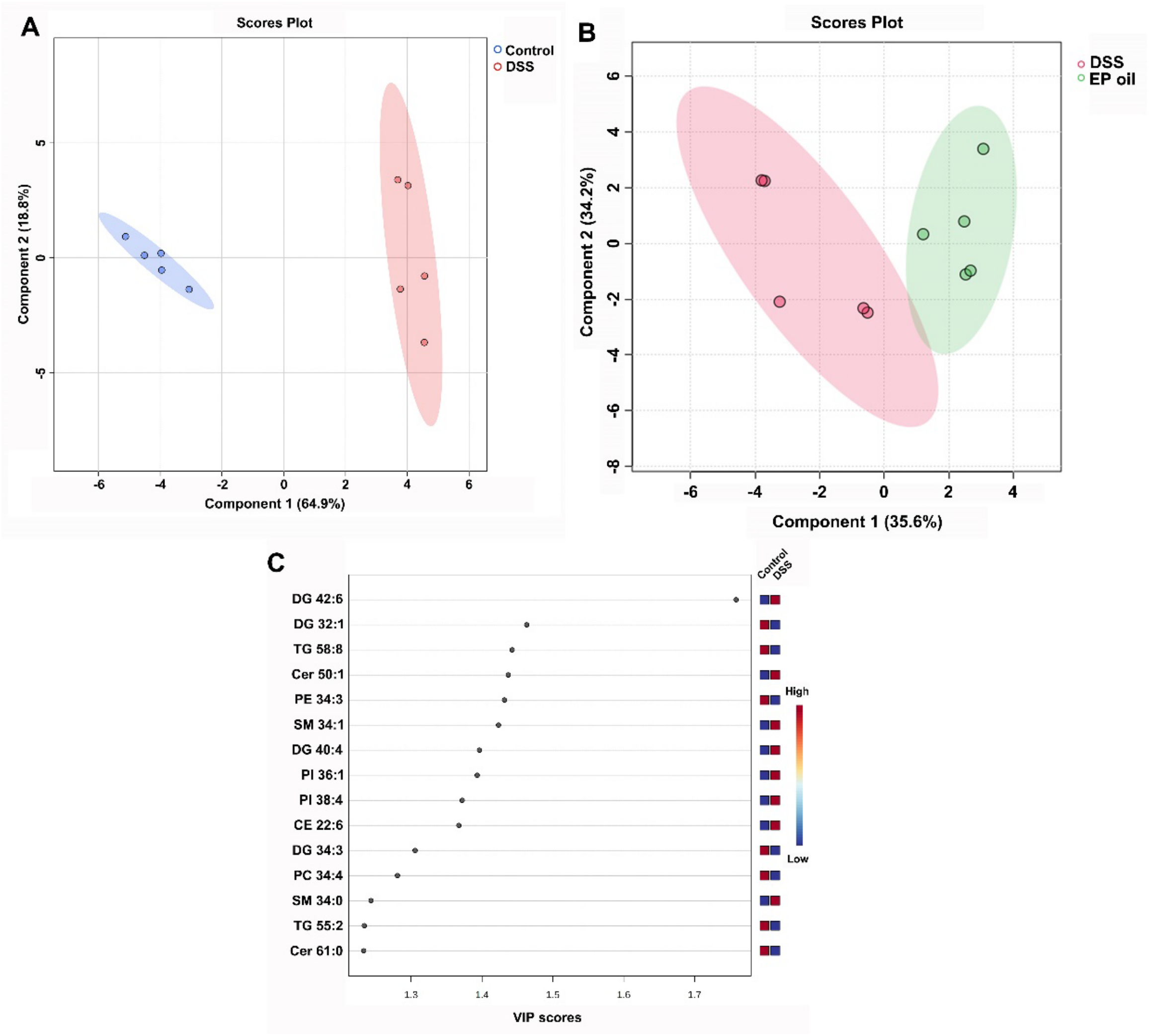

Figure S2 Heat map of (A) control and DSS groups and (B) EP oil and DSS group.

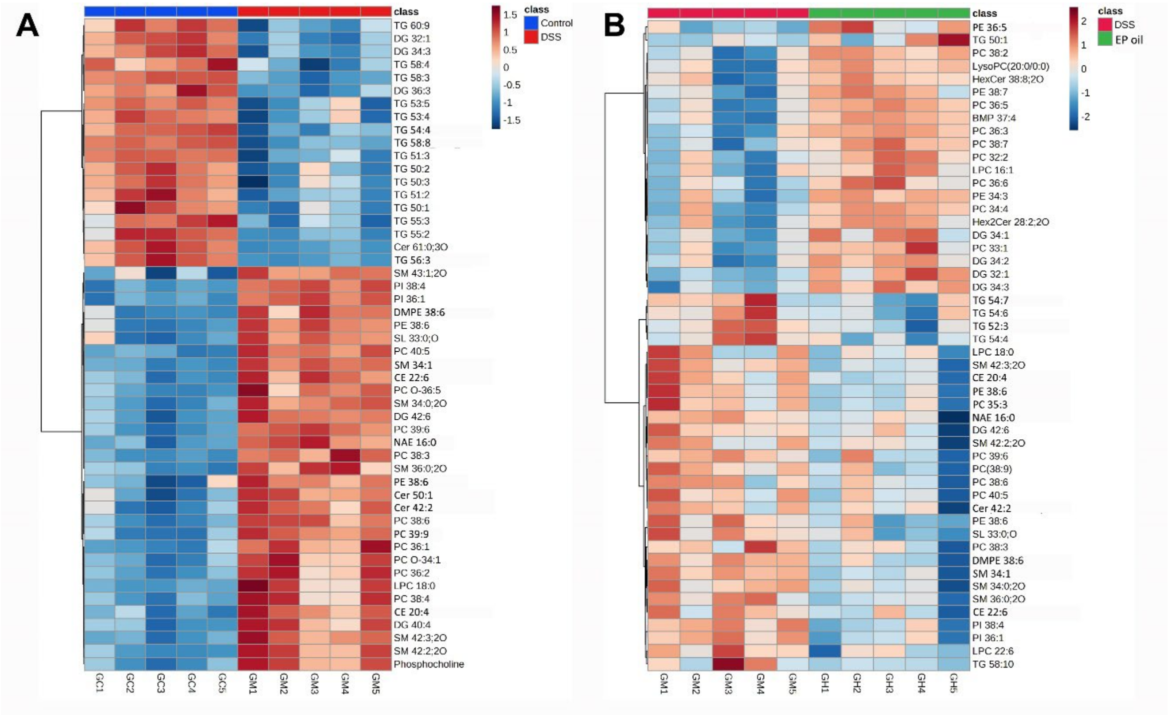

Figure S3 Volcano plot of (A) control and DSS groups and (B) EP oil and DSS group.

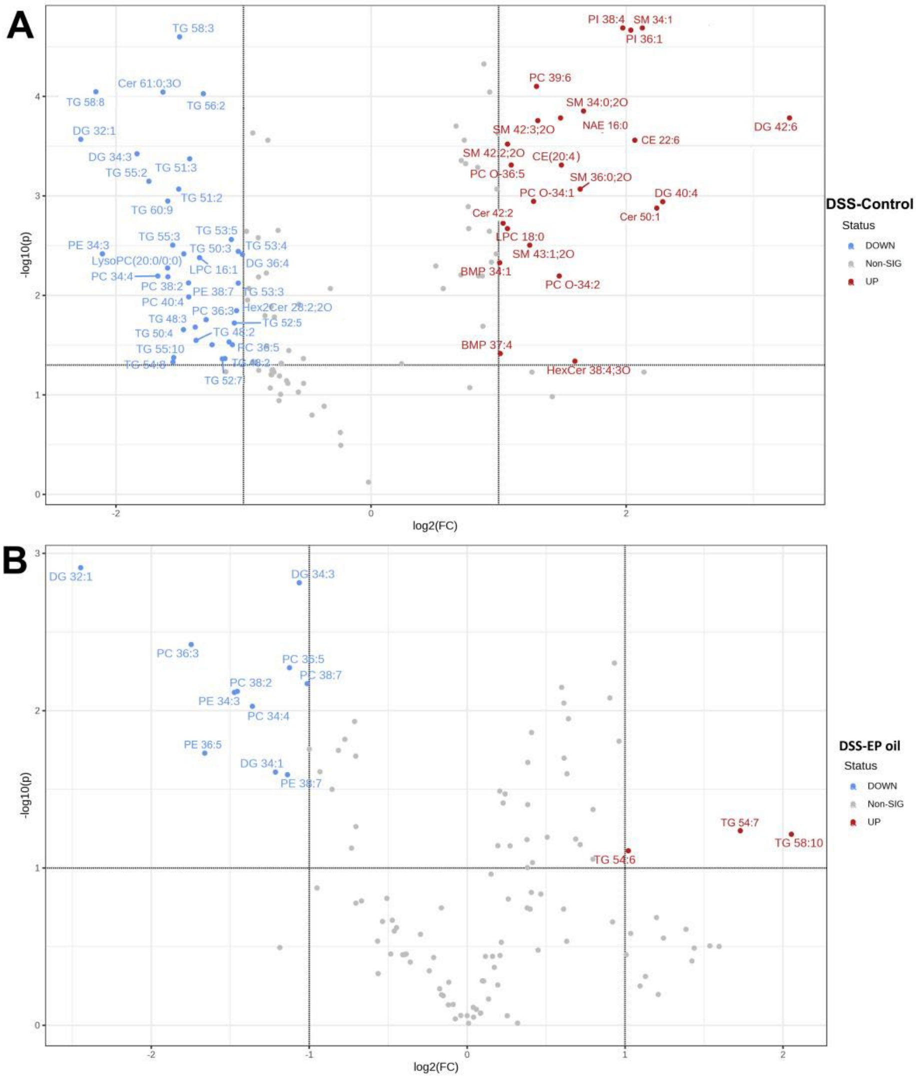

Figure S4 Lipid metabolite enrichment analysis between control and DSS groups.

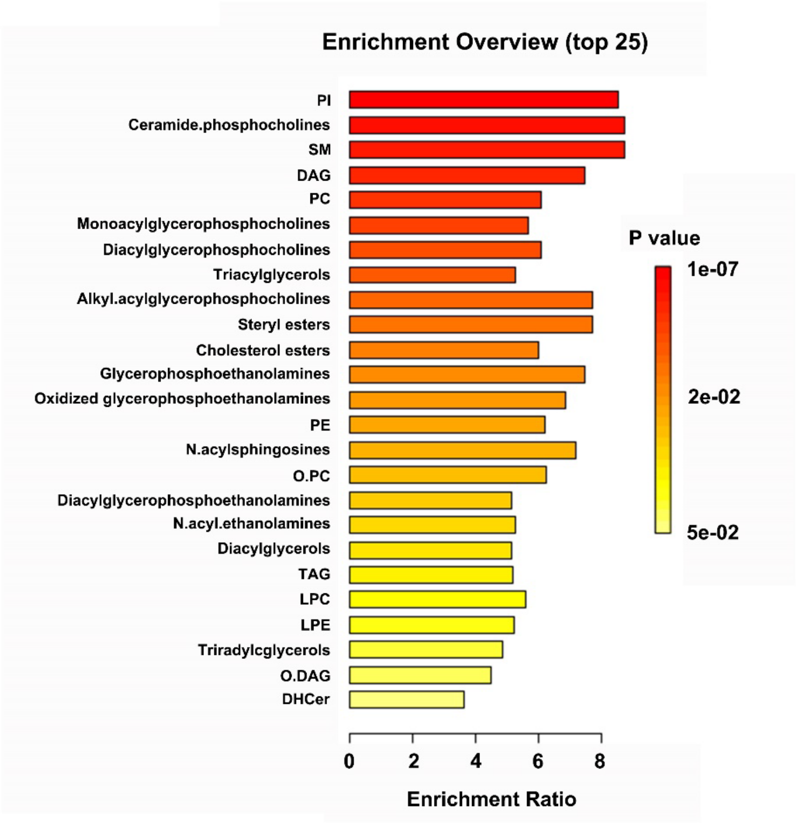

Figure S5 Metabolism pathway analysis of lipid metabolites between control and DSS groups.

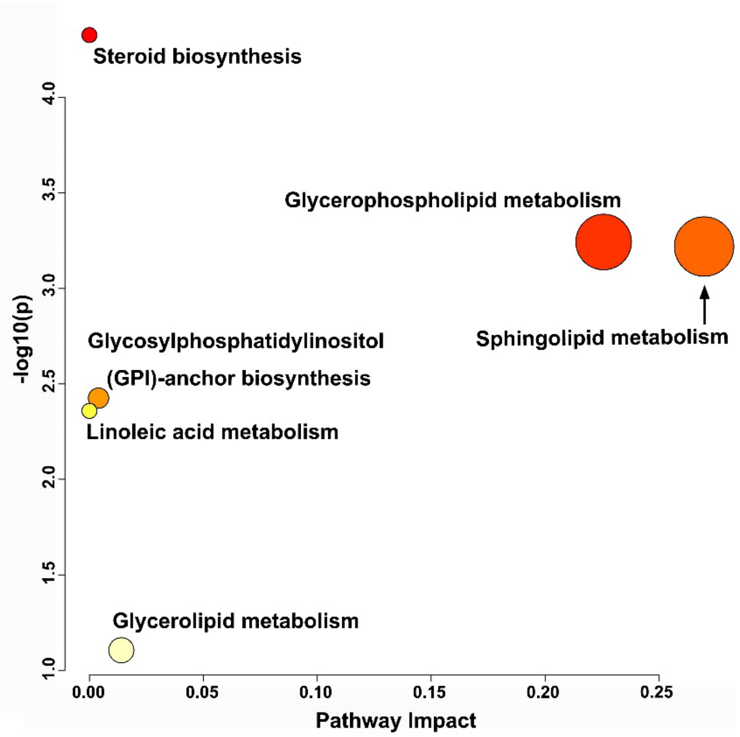

Supplement: Supplementary file 1 [file foods-13-00046-s001.zip › foods-2761020-supplementary.pdf]
